# Case report: Intrapulmonary tidal volumes in a preterm infant with chest wall rigidity

**DOI:** 10.3389/fped.2022.979763

**Published:** 2022-08-23

**Authors:** Vincent D. Gaertner, Tanja Restin, Dirk Bassler, Jean-Claude Fauchère, Christoph M. Rüegger

**Affiliations:** Newborn Research, Department of Neonatology, University Hospital Zürich, University of Zürich, Zürich, Switzerland

**Keywords:** chest wall rigidity, electrical impedance tomography, preterm infant, wooden chest syndrome, endotracheal intubation, case report

## Abstract

**Background:**

Chest wall rigidity is a known side effect of fentanyl use, which is why fentanyl is usually combined with a muscle relaxant such as mivacurium. Verifying endotracheal intubation is difficult in case of a rigid chest wall.

**Case presentation:**

We present the case of a preterm infant (29 completed weeks gestation, birth weight 1,150 g) with a prolonged chest wall rigidity after fentanyl administration for intubation despite adequate doses of mivacurium. This resulted in a pronounced desaturation without any effect on heart rate. Clinically, the infant showed no chest wall movement despite intubation and common tools to verify intubation (including end-tidal carbon dioxide measurement and auscultation) were inconclusive. However, using electrical impedance tomography (EIT), we were able to demonstrate minimal tidal volumes at lung level and thereby, EIT was able to accurately show correct placement of the endotracheal tube.

**Conclusions:**

This case may increase vigilance for fentanyl-induced chest wall rigidity in the neonatal population even when simultaneously administering mivacurium. Higher airway pressures exceeding 30 mmHg and the use of μ-receptor antagonists such as naloxone should be considered to reverse opioid-induced chest wall rigidity. Most importantly, our data may imply a relevant clinical benefit of using EIT during neonatal intubation as it may accurately show correct endotracheal tube placement.

## Background

Chest wall rigidity is a known side effect of fentanyl use, mostly with higher doses administered rapidly (1, 2). While it has been described in the neonatal (3), pediatric (4), and adult population (1, 5), known cases received no muscle relaxant which could circumvent this side effect (6). We present the case of a preterm infant with presumed prolonged chest wall rigidity after fentanyl administration for intubation despite adequate doses of mivacurium, and we demonstrate the development of intrapulmonary volume changes during the entire episode.

## Case presentation

The patient participated in a recent randomized controlled trial investigating the effect of surfactant nebulization (clinicalTrials.gov identifier: NCT04315636). We continuously recorded heart rate (HR) and preductal oxygen saturation (SpO_2_) (Masimo Radical 7; Masimo Corporation, Irvine, USA) as well as electrical impedance tomography (EIT) data (LuMon^TM^; SenTec AG, Landquart, Switzerland). EIT data was analyzed as previously described (7, 8). Airway flow and pressure were measured using a flow sensor at the Y-piece of the nasopharyngeal/endotracheal tube and recorded at 200 Hz (NewLifeBox^®^, Advanced Life Diagnostics UG, Weener, Germany) (9). Resuscitations were video recorded from above. Mean values and 95% confidence intervals (95% CI) were calculated over ten seconds at each time point.

After a pregnancy with prolonged premature rupture of membranes and anhydramnios for four weeks, the infant was delivered *via* cesarean section at 29^3/7^ weeks gestation with a birth weight of 1,150 g. Antenatal steroid prophylaxis was completed before birth. The infant was initially bradypneic and was stabilized using non-invasive positive pressure support (NIPPV). Due to persistent bradycardia despite increased positive airway pressures, a nasopharyngeal tube was inserted 5 min after birth upon which HR normalized. The infant had an Apgar score of 8, 8, and 9 at 1, 5, and 10 min after birth. However, the infant continued to require a FiO_2_ of 0.30-0.40 to achieve preductal SpO_2_ of 87-95% and showed insufficient ventilation [increase of partial pressure of carbon dioxide (pCO_2_) from 8.8 kPa to 10.4 kPa over 20 min]. Due to global respiratory insufficiency, the decision was made to intubate the infant.

**Figure 1:**
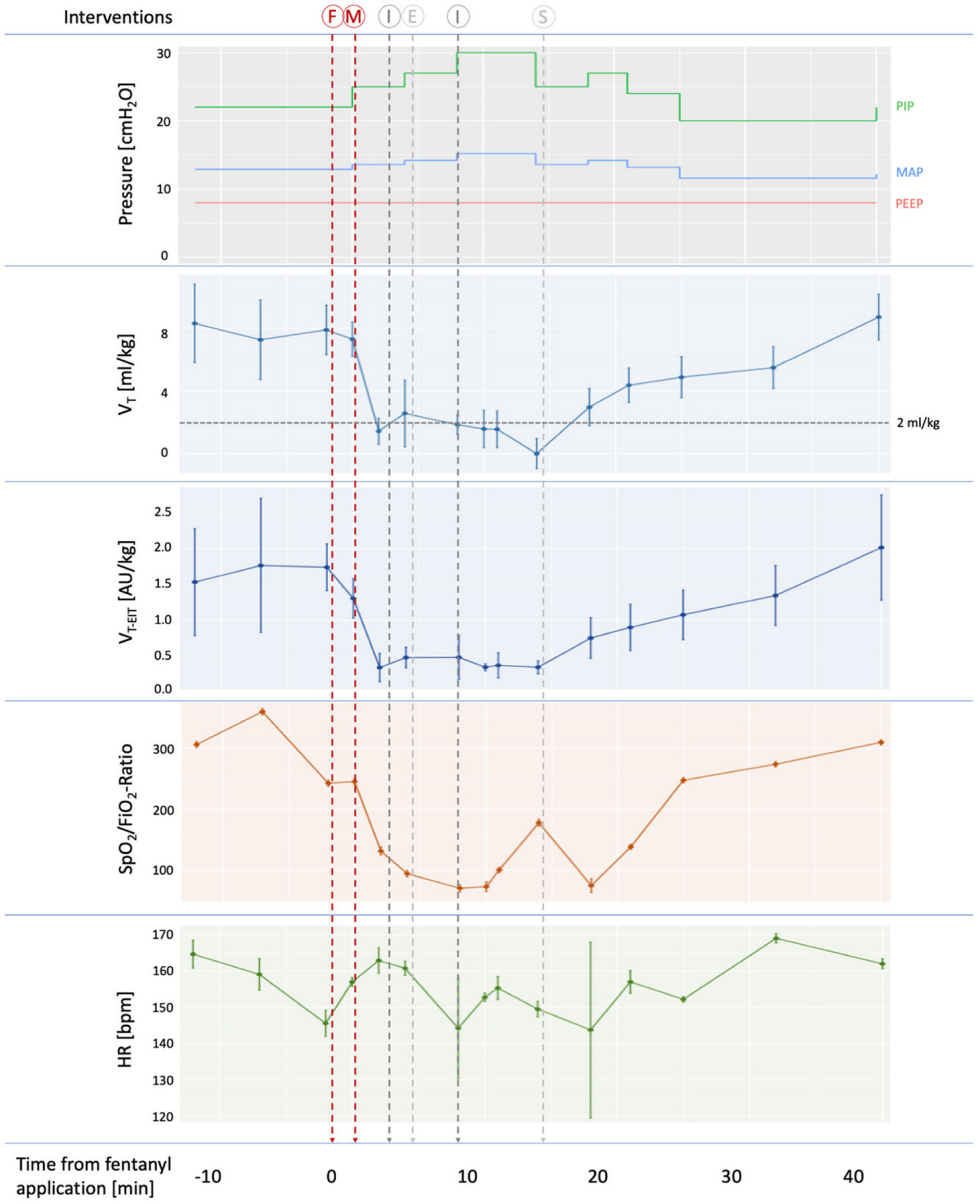
Development of cardiorespiratory parameters during the intubation procedure. Applied pressures, as well as measured tidal volumes, oxygenation and heart rate are shown. Tidal volumes were measured in the ventilator circuit using a flow sensor at the proximal end of the endotracheal tube (*V*_*T*_) and intrapulmonary *V*_*T*_ were measured using electrical impedance tomography (*V*__*T*_−EIT_). Mean values and 95% confidence intervals of ten-second intervals for each time point are shown. Insufficient ventilation (*V*_*T*_ < 2ml/kg) is indicated by a dotted horizontal line. Timing is provided in minutes relative to fentanyl application. Vertical lines indicate interventions performed: F, fentanyl administration; M, mivacurium administration; I, intubation; E, extubation; S, surfactant administration. PIP, peak inspiratory pressure, MAP, mean airway pressure, PEEP, positive end-expiratory pressure.

Fentanyl (1μg/kg) was administered *via* peripheral intravenous access over 40 s for analgosedation followed by a slow infusion of a fluid bolus, and mivacurium (0.2 mg/kg) for muscle relaxation. The infant was intubated 62 min after birth. After intubation, the infant rapidly desaturated and required a FiO_2_ of 1.0. Therefore, misplacement of the endotracheal tube was assumed and the infant was extubated and re-intubated. Still, no color change on the end-tidal CO_2_ detector (etCO_2_) was observed although the vocal cords were visible and ETT depth at the lip was visually checked by the treating physicians during both intubation attempts. Leakage was minimal (ranging between 0 and 20%) after each intubation attempt. As the desaturation persisted even after increasing peak inspiratory pressures, the appropriate positioning of the endotracheal tube (ETT) was independently confirmed *via* laryngoscopy by a senior attending and subsequently, surfactant was administered intratracheally 74 min after birth. Slowly, oxygenation improved, V_T_ increased slightly and the capnometer started to change color. All values were returned to pre-medication values approximately 35 min after administration of fentanyl and mivacurium. Throughout the entire episode, the infant required a FiO_2_ of 0.8–1.0 and achieved preductal SpO_2_ values of 70–95%, and measured *V*_*T*_ was <2 ml/kg without relevant leak, indicative of airway obstruction or low respiratory system compliance. Heart rate was not significantly affected. The development of cardiorespiratory parameters is depicted in Figure 1. At lung level, *V*_*T*_ were detectable using EIT (indicating correct placement of the ETT) but only minimal amplitudes were noted (see Supplementary Video).

Two and a half hours after birth, the infant developed a right-sided pneumothorax which was relieved immediately and resolved within 24 h. Severe respiratory acidosis was persistent (venous blood gas analysis 3 h after intubation: pH 7.12 and pCO_2_ 12.1 kPa) and the infant was switched to high-frequency oscillatory ventilation (HFOV) which quickly improved ventilation. The infant was extubated on day four, received respiratory support using nasal CPAP and high flow until day 42 after birth. He was discharged after 57 days at 37 ^3/7^ weeks post-menstrual age with a mild bronchopulmonary dysplasia (Walsh criteria). On day 30, blood was drawn for further investigation of the incident. Cholinesterase activity was 5.5 kU/l (Ref: 5.3–12.9) and the dibucaine number was 0.76 (Ref: >0.72).

## Discussion and conclusion

We describe a case of pronounced chest wall rigidity in a very preterm neonate after application of fentanyl for analgosedation despite the simultaneous use of mivacurium. To our knowledge, this is the first report of intrapulmonary volume changes during chest wall rigidity.

Chest wall rigidity is a known phenomenon after fentanyl application (1–3), even after prenatal exposure (10). Muscle relaxants can reduce this phenomenon (6), and thus, mivacurium was suggested as an appropriate addition to fentanyl before neonatal intubation (11). Mivacurium is metabolized by plasma cholinesterase (pCHE) and increased levels of pCHE are associated with a rapid inactivation of mivacurium (6). In the presented case, pCHE activity and dibucaine number were within the reference range, indicating a regular metabolism of mivacurium (6). Still, chest wall rigidity was pronounced with a prolonged phase of desaturation, possibly due to the rapid application of fentanyl. The infant remained normocardic and we speculate that minimal tidal volumes reaching the lung may have been sufficient to prevent bradycardia for this time period. While we already increased peak inspiratory pressures, this may still have been insufficient, indicated by the still minimal tidal volumes measured by respiratory function monitor as well as by EIT. Other cases report the necessity to increase peak pressures in excess of 50 mmHg in case of persistent hypoventilation despite correct intubation (12, 13).

There are various potential differential diagnoses for the observations made in this case which presented with severely low dynamic lung compliance or airway obstruction. Most notably, functional lung hypoplasia after PPROM and concomitant oligohydramnios may result in a transiently poor lung compliance (i.e., “dry lung”) and consequent hypoventilation, as was the case in this infant. Other causes such as surfactant deficiency, secretion-related airway obstruction, equipment failure, pneumothorax or one-sided bronchial intubation need to be considered in newborns who are difficult to ventilate. While the marked and steep decrease in V_T_ after the application of fentanyl (and before intubation) are unlikely in most of these differential diagnoses, the persistent hypercarbia 3 h after the incident implies that oligohydramnios-related lung hypoplasia may have played an additional role in the pathophysiology of the event. Thus, we speculate, that there was an interplay between various factors (i.e., chest wall rigidity and oligohydramnios-related functional lung hypoplasia) explaining the short- and long-term course of this patient.

The common tools to verify correct ETT placement (e.g., thoracic excursions, visual laryngoscopic inspection, auscultation and etCO_2_) are inconclusive in case of a rigid chest wall as tidal volumes are insufficient for thoracic movement or gas exchange (and concomitant color change on a capnometer). By using EIT, we could retrospectively visualize tidal excursions on lung level, and we speculate that EIT could be prospectively used to differentiate intratracheal ETT placement from esophageal position during neonatal intubation as has been suggested in a small animal study (14). Possibly, combining the information from EIT and respiratory function monitor (i.e., leakage, etCO_2_) may be ideal in determining correct intubation. However, further studies are needed.

This report highlights (1) the need to remain vigilant when using fentanyl for neonatal intubation despite the simultaneous use of muscle relaxants, and (2) the importance of a slow application of fentanyl as well as the subsequent flush. Also, as tidal volumes on lung level were minimal, clinicians should consider increasing airway pressures until a perceptible chest rise is noted, possibly exceeding 50 mmHg. The risk of volu- or barotrauma is likely minimal until adequate tidal volumes are administered. Also, μ-receptor antagonists such as naloxone are required in cases of opioid-related chest wall rigidity and having the dose pre-calculated in the intubation set may minimize delays. Finally, our data may imply that using EIT may yield relevant clinical information during (neonatal) intubation.

## Data availability statement

The datasets presented in this article are not readily available because due to containing information that could compromise the privacy of the patient. Data is available from the corresponding author (VG) upon reasonable request.

## Ethics statement

The studies involving human participants were reviewed and approved by Cantonal Ethics Committee of Zürich (Approval Number KEK-2020-00890). Written informed consent to participate in this study was provided by the participants' legal guardian/next of kin. Written informed consent was obtained from the minor(s)' legal guardian/next of kin for the publication of any potentially identifiable images or data included in this article.

## Author contributions

VG collected data, analyzed the data, and wrote the first draft of the manuscript. TR, J-CF, DB, and CR contributed to redrafting the manuscript and revising it for intellectual input. CR supervised the project. All authors read and approved the final manuscript.

## Funding

This original study was sponsored by the EMDO Foundation, the European Society for Pediatric Research, the Heuberg-Foundation and the SwissLife Foundation. The funders had no role in data collection, data analysis, data interpretation, and manuscript writing.

## Conflict of interest

The authors declare that the research was conducted in the absence of any commercial or financial relationships that could be construed as a potential conflict of interest.

## Publisher's note

All claims expressed in this article are solely those of the authors and do not necessarily represent those of their affiliated organizations, or those of the publisher, the editors and the reviewers. Any product that may be evaluated in this article, or claim that may be made by its manufacturer, is not guaranteed or endorsed by the publisher.

## Abbreviations

EIT: Electrical impedance tomography
etCO_2_: End-tidal CO_2_ detector
HFOV: High-frequency oscillatory ventilation
HR: Heart rate
NIPPV: Non-invasive positive pressure support
pCHE: Plasma cholinesterase
pCO_2_: Partial pressure of carbon dioxide
SpO_2_: Peripheral oxygen saturation
V_T_: Tidal volumes as measured by flow sensor
V_T−EIT_: Intrapulmonary tidal volumes as measured using EIT.
